# How does CBCT reconstruction algorithm impact on deformably mapped targets and accumulated dose distributions?

**DOI:** 10.1002/acm2.13328

**Published:** 2021-08-10

**Authors:** Weihua Mao, Chang Liu, Stephen J. Gardner, Mohamed Elshaikh, Ibrahim Aref, Joon K. Lee, Deepak Pradhan, Farzan Siddiqui, Karen Chin Snyder, Akila Kumarasiri, Bo Zhao, Joshua Kim, Haisen Li, Ning Winston Wen, Benjamin Movsas, Indrin J. Chetty

**Affiliations:** ^1^ Henry Ford Health System Detroit MI USA

**Keywords:** deformable dose accumulation, iCBCT

## Abstract

**Purpose:**

We performed quantitative analysis of differences in deformable image registration (DIR) and deformable dose accumulation (DDA) computed on CBCT datasets reconstructed using the standard (Feldkamp‐Davis‐Kress: FDK_CBCT) and a novel iterative (iterative_CBCT) CBCT reconstruction algorithms.

**Methods:**

Both FDK_CBCT and iterative_CBCT images were reconstructed for 323 fractions of treatment for 10 prostate cancer patients. Planning CT images were deformably registered to each CBCT image data set. After daily dose distributions were computed, they were mapped to planning CT to obtain deformed doses. Dosimetric and image registration results based CBCT images reconstructed by two algorithms were compared at three levels: (A) voxel doses over entire dose calculation volume, (B) clinical constraint results on targets and sensitive structures, and (C) contours propagated to CBCT images using DIR results based on three algorithms (SmartAdapt, Velocity, and Elastix) were compared with manually delineated contours as ground truth.

**Results:**

(A) Average daily dose differences and average normalized DDA differences between FDK_CBCT and iterative_CBCT were ≤1 cGy. Maximum daily point dose differences increased from 0.22 ± 0.06 Gy (before the deformable dose mapping operation) to 1.33 ± 0.38 Gy after the deformable dose mapping. Maximum differences of normalized DDA per fraction were up to 0.80 Gy (0.42 ± 0.19 Gy). (B) Differences in target minimum doses were up to 8.31 Gy (−0.62 ± 4.60 Gy) and differences in critical structure doses were 0.70 ± 1.49 Gy. (C) For mapped prostate contours based on iterative_CBCT (relative to standard FDK_CBCT), dice similarity coefficient increased by 0.10 ± 0.09 (*p* < 0.0001), mass center distances decreased by 2.5 ± 3.0 mm (*p* < 0.00005), and Hausdorff distances decreased by 3.3 ± 4.4 mm (*p* < 0.00015).

**Conclusions:**

The new iterative CBCT reconstruction algorithm leads to different mapped volumes of interest, deformed and cumulative doses than results based on conventional FDK_CBCT.

## 1 INTRODUCTION

Current radiation therapy techniques enable accurate planning of external beam radiotherapy treatment,^1^, ^2^ but it typically involves dose calculation on one image set—the simulation CT. For many disease sites, however, changes in patient anatomy (e.g., due to weight loss, tumor response, and other organ movement) are common and patient positioning may be different from treatment to treatment.^3^, ^4^, ^5^, ^6^, ^7^, ^8^, ^9^ These changes in geometry of both the target and normal tissues impact the delivered dose distribution. With daily 3D imaging of these patients, physicians have an opportunity to evaluate the need for a re‐simulation daily—and thus to re‐start the planning process from scratch with a new set of images. Of course, if a new plan is generated, then the subsequent treatments are likely to be more accurately tailored to the patient's present anatomy, but one question remains unanswered—what is the true total dose delivered to the patient throughout the course of treatment? Deformable dose accumulation (DDA) attempts to answer the above question by generating a geometric mapping between two image sets using deformable image registration (DIR). In short, the process of DDA includes generation of daily volumetric images, daily dose calculation, daily delivery evaluation, and deformation of dose to reference image set to obtain cumulative dose. DIR can be used as follows: (1) To deform planning CT to daily positioning cone‐beam computed tomography (CBCT) or other online volumetric images to calculate delivered dose daily; (2) To then deform planning contoured volume of interest (ROI) to the daily images for daily dose evaluation; and (3) To map daily delivered dose to the reference images (planning CT) to obtain cumulative dose. It has been reported that the performance of soft tissue DIR may be affected by imaging quality.^10^, ^11^, ^12^

Recently, a novel iterative CBCT (iterative_CBCT) reconstruction algorithm has become available in clinic. In addition to a standard kernel‐based correction followed by filtered back‐projection‐based CBCT (FDK_CBCT), iterative_CBCT uses a finite element solver (AcurosCTS)‐based scatter correction and a statistical (iterative) reconstruction.^13^, ^14^, ^15^ Noise and contrast‐to‐noise ratio image quality characteristics were improved using the iterative_CBCT reconstruction algorithm relative to the FDK_CBCT method using in‐phantom methods^16^ and clinical patient datasets.^17^ In this study, we compare the differences in DDA results and DIR results performed with different algorithms on CBCT datasets reconstructed by standard (FDK) vs. iterative reconstruction techniques.

## 2 METHODS AND MATERIALS

**FIGURE 1:**
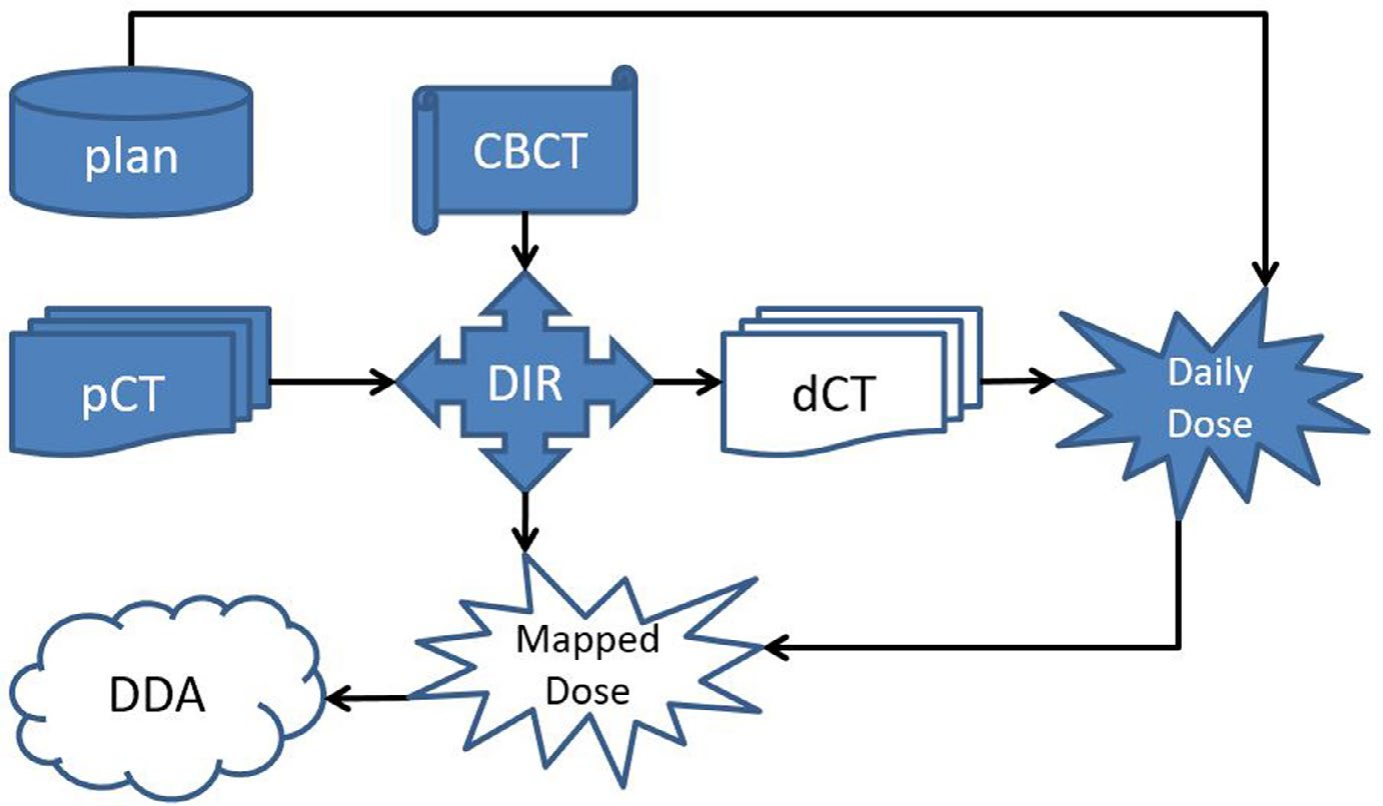
Flowchart of the in‐house deformable dose accumulation (DDA) analysis. Planning CT(pCT) was deformably registered to CBCT to generate deformed CT (dCT). Dailydose was calculated on dCT and then mapped to pCT using the same deformable imageregistration (DIR) results. DDA was the summation of all mapped dailydoses.

A total of 10 prostate cancer patients were randomly selected from a 6‐month time window for an IRB‐approved retrospective analysis. Three types of treatments were prescribed, 2.00 Gy per fraction by 39, 1.80 Gy per fraction by 44 fractions, and 1.80 Gy per fraction by 25 fractions. Two‐Arc VMAT plans were generated for each patient in Eclipse (Varian Medical Systems) and treated using Truebeam linear accelerators (Varian Medical Systems). Daily CBCT images were acquired to position patients for treatment in the study. Using the same raw data of each daily CBCT image set, both FDK_CBCT and iterative_CBCT images were reconstructed for 323 total treatment fractions. The DDA procedure was performed for FDK_CBCT and iterative_CBCT images; (see Figure 1 for details of the DDA procedure). For each CBCT reconstruction algorithm type, planning CT (pCT) images were deformed to CBCT images according to the following steps:

1. Rigid alignment between pCT and CBCT using treatment positions from treatment records.
2. DIR of pCT to CBCT, accounting for the corresponding volume of pCT to the volume of CBCT since the pCT usually has a much larger field‐of‐view (FOV) than that of CBCT images. An open source software, Elastix, was used for DIR.^18^, ^19^ Elastix models anatomic deformations using a b‐spline algorithm with a well‐defined global smoothness parameter. Four registration steps are included, using translational, Euler, affine, and B‐spline transformations sequentially. Mutual information is used as the similarity measurement for all registrations. This portion of pCT was deformed to the volume of CBCT.
3. The remaining volume of the pCT was outside of the CBCT FOV and was stitched to the deformed part of the pCT directly to generate the entire deformed CT (dCT). The final complete transformation map or deformable vector field (DVF) was saved.

Daily delivered dose distribution was calculated on the dCT in Eclipse using identical dose calculation algorithms and parameters of the original reference plans. Daily dose distributions were mapped to planning CT using the final transformation map and DDA was the summation of mapped daily dose distribution over a treatment course. The effects of CBCT image quality on DIR and DDA were investigated as follows: (A) voxel dose differences in the entire dose calculation volume, (B) dose differences of clinical constraints: high dose region or target and sensitive structures; (C) DIR results differences based on mapped contours.

### 2.1 Voxel dose differences between results based on FDK_CBCT and iterative_CBCT images in the entire dose calculation volume

Three comparisons were conducted between dose distributions based on FDK_CBCT and iterative_CBCT:

1. Daily doses on dCT before dose mapping were compared by calculating image‐voxel‐based average, standard deviation, and maximum dose differences in CBCT volumes.
2. Daily doses on pCT after dose mapping were compared by calculating image‐voxel‐based average, standard deviation, and maximum dose differences in CBCT volumes.
3. Normalized DDA or DDA per day, was calculated by dividing the total DDA by number of treatment fractions. Normalized DDA values were compared by calculating image‐voxel‐based average, standard deviation, and maximum normalized DDA differences in CBCT.

### 2.2 Dose differences of clinical constraints

Clinical goals were evaluated by comparing dosimetric constraint results for targets and critical structures using DDA based on FDK_CBCT and iterative_CBCT images. Maximum dose (Dmax), minimum dose (Dmin), and dose level covering 95% of the volume (D95%) were evaluated for both PTV and clinical target volume (CTV). The minimum and maximum points were defined as 0.035 cc instead of a single voxel. Average dose (Dmean) was evaluated for PTV. Clinical constraints for organs at risk depended on the protocol used. Generally, Dmean of rectum, bladder, and penile bulb were evaluated. Doses covering 5% of volume (D5%) were evaluated for femoral heads.

### 2.3 Impact of CBCT reconstruction on DIR accuracy

Three DIR algorithms were used to register planning CT to one pair of CBCT images for each prostate patient: Elastix (B‐spline‐based, open source) and two commercially available algorithms, SmartAdapt (Demons‐based, Eclipse/Varian Medical Systems, Palo Alto, CA) and Velocity (B‐spline‐based, Varian).

Manual contouring was performed in ARIA (Varian Medical Systems). Three to five physicians contoured one pair of CBCT images for each of 10 prostate patients. Four structures, prostate gland alone, proximal 2 cm seminal vesicles (SV), rectum, and bladder, were delineated on both sets of CBCT images blindly. These observers contoured patient datasets one patient at a time and were blinded to the image set reconstruction mode (the image sets were labeled with ‘_A’ and ‘_B’ for each patient to represent the FDK and Iterative data sets—these labels were randomly assigned for each patient). The following guidance was provided for contouring: the rectum borders are defined inferiorly as the pubic ramus and ischial tuberosity and superiorly as flexure of sigmoid colon. For each structure type, a consensus contour was generated using the simultaneous truth and performance level estimation (STAPLE) method in the Computational Environment for Radiotherapy Research (CERR) software package.^20^, ^21^

Three metrics were used to evaluate agreement between mapped contours and consensus contours. The Dice similarity coefficient (DSC) was used to evaluate general overlap between mapped contours and consensus contours^22^:

(1) $$\text{DSC =}\;\frac{2\left|V_{\text{map}}\;\cap \;V_{\text{cons}}\right|}{\left|V_{\text{map}}\right|\;+\;\left|V_{\text{cons}}\right|}$$

$V_{\mathrm{map}}$ is mapped contour from planning CT to CBCT and $V_{\mathrm{cons}}$ is the corresponding consensus contour on the same set of CBCT images. DSC difference was defined as the DSC between contours mapped to iterative_CBCT and consensus contours on the iterative_CBCT subtracted by the DSC between contours mapped to FDK_CBCT and consensus contours on the FDK_CBCT:

(2) $$\text{DSC difference}\;=\;\text{DSC}\left(V_{\text{map}\_\text{iterative}},{\;V}_{\text{cons}\_\text{iterative}}\right)\;‐\;\text{DSC}\left(V_{\text{map}\_\text{FDK}},{\;V}_{\text{cons}\_\text{FDK}}\right)$$

V_map_iterative_ and V_cons_iterative_ are mapped contour and consensus contour based on iterative CBCT images, respectively. V_map_FDK_ and V_cons_FDK_ are mapped contour and consensus contour based on FDK CBCT images respectively.

The mean contour distance (MCD) was the distance between centers of mass of mapped and consensus contours. MCD difference was defined as the MCD between contours mapped to iterative_CBCT and consensus contours on the iterative_CBCT subtracted by the MCD between contours mapped to FDK_CBCT and consensus contours on the FDK_CBCT:

(3) $$\text{MCD difference}\;=\;\text{MCD}\left(V_{\text{map}\_\text{iterative}},{\;V}_{\text{cons}\_\text{iterative}}\right)\;‐\;\text{MCD}\left(V_{\text{map}\_\text{FDK}},{\;V}_{\text{cons}\_\text{FDK}}\right)$$

Hausdorff distance (HD) was used to evaluate gross error between the mapped and consensus contours. The Hausdorff distance (HD) was the maximum distance of a point in one contour to the nearest point of the other contour and is defined as:

(4) $$\text{HD}\left(V_{\text{map}},V_{\text{cons}}\right)={\text{max}}_{a\in V_{\text{map}}}\left\{{\text{min}}_{b\in V_{\text{cons}}}\left\{d\left(a,b\right)\right\}\right\}$$

where *a* and *b we*re points of contours $V_{\mathrm{map}}$ and $V_{\mathrm{cons}}$, respectively, and *d*(*a*, *b*) was Euclidean metric between these points.^23^, ^24^ HD difference was defined as the HD between contours mapped to iterative_CBCT and consensus contours on the iterative_CBCT subtracted by the HD between contours mapped to FDK_CBCT and consensus contours on the FDK_CBCT:

(5) $$\text{HD difference}\;=\;\text{HD}\left(V_{\text{map}\_\text{iterative}},{\;V}_{\text{cons}\_\text{iterative}}\right)\;‐\;\text{HD}\;\left(V_{\text{map}\_\text{FDK}},{\;V}_{\text{cons}\_\text{FDK}}\right)$$

Statistical analyses of DSC difference, MCD difference, and HD difference were performed using Student's *t*‐test (1 tail, *p* < 0.05 significant).

## 3 RESULTS

### 3.1 Dose differences in the entire dose calculation volume

**FIGURE 2:**
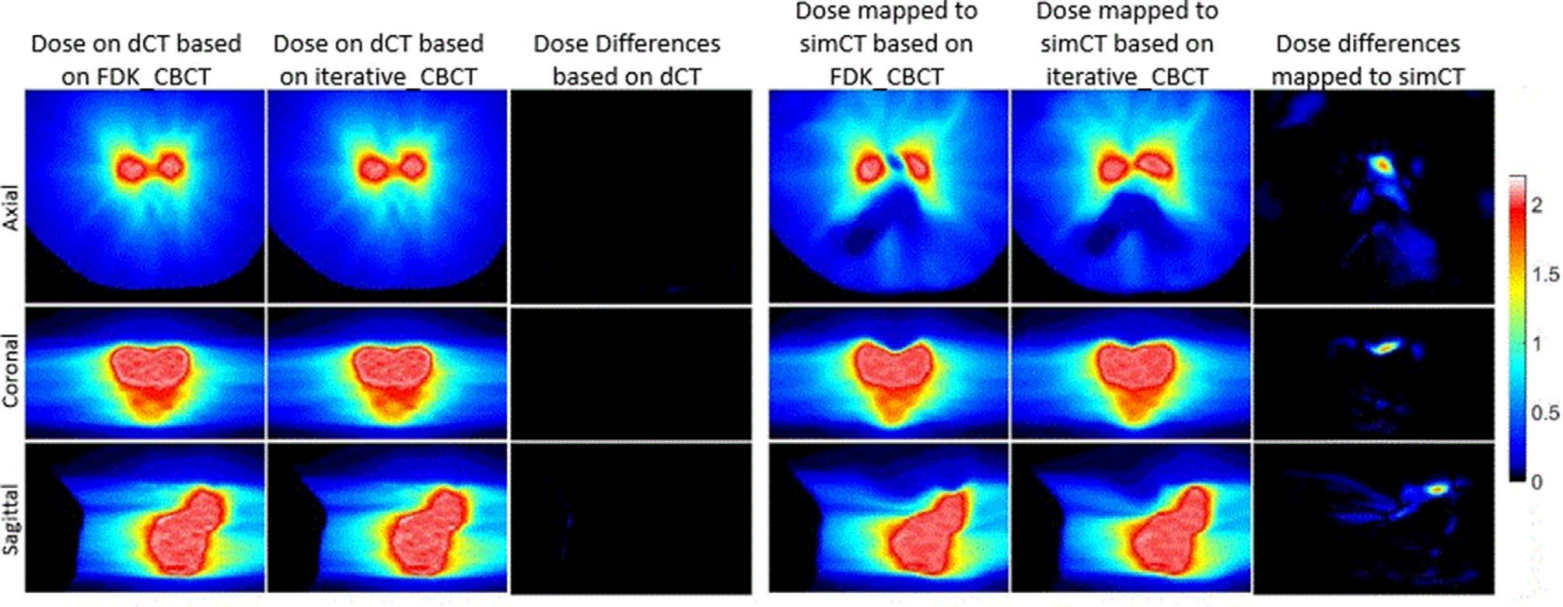
Axial (top row), coronal (middle row), and sagittal (lower row) views of dose distribution comparisons of a single fraction of treatment before and after dose mapping based on two reconstruction algorithms and their differences (Unit: Gy).

Daily dose distributions were calculated for every fraction of treatment based on FDK_CBCT and iterative_CBCT images. Differences were analyzed using the image voxel‐based dose subtraction. Figure 2 compares dose distributions of a single fraction of treatment before and after dose mapping based on each CBCT reconstruction algorithm and their differences (direct subtraction). Differences of dose distributions between two CBCT algorithms are compared in Table 1. The overall daily differences and normalized DDA differences between results from FDK_CBCT and iterative_CBCT were 0.00 ± 0.01 Gy for 323 fractions of treatments of 10 patients. As highlighted in Table 1, patient PC09 has a maximum daily dose differences of 1.66 Gy after dose mapping and normalized DDA differences of 0.80 Gy. Patient PC30 has a maximum daily dose differences of 1.73 Gy after dose mapping and normalized DDA differences of 0.25 Gy. For all patients, maximum daily point dose differences between results from two reconstruction algorithms increased from 0.22 ± 0.06 Gy before dose mapping to 1.33 ± 0.38 Gy after dose mapping, and maximum normalized DDA differences were 0.42 ± 0.19 Gy over CBCT volumes. Maximum dose differences occurred in the high dose gradient region in the pelvis.

**TABLE 1:** List of dose comparison between results based on FDK_CBCT and iterative_CBCT images

Patient #	# of Fractions	Daily Dose Differences Before Dose Mapping (Gy)			Daily Dose Differences After Dose Mapping (Gy)			Normalized DDA Differences (Gy)		
		Avg	Stdev	Max	Avg	Stdev	Max	Avg	Stdev	Max
PC09	26	0.00	0.00	0.13	0.00	0.02	1.66	0.00	0.01	0.80
PC12	36	0.00	0.00	0.28	0.00	0.02	1.60	0.00	0.01	0.33
PC13	38	0.00	0.04	0.25	0.00	0.01	0.58	0.00	0.00	0.15
PC16	34	0.00	0.03	0.22	0.00	0.02	1.43	0.00	0.01	0.46
PC17	34	0.00	0.04	0.16	0.00	0.02	1.67	0.00	0.01	0.45
PC22	20	0.00	0.03	0.26	0.00	0.01	0.84	0.00	0.01	0.26
PC25	39	0.00	0.01	0.16	0.00	0.01	1.24	0.00	0.01	0.48
PC28	25	0.00	0.00	0.21	0.00	0.01	1.32	0.00	0.01	0.65
PC30	30	0.00	0.05	0.32	0.00	0.01	1.73	0.00	0.00	0.25
PC31	41	0.00	0.02	0.18	0.00	0.01	1.22	0.00	0.01	0.36

For every fraction of treatment, average (Avg), standard deviation (Stdev), and maximum (Max) dose differences were calculated. The listed Avg of daily dose differences is the average over of every fraction average of dose differences; the listed Stdev of daily dose differences is the average of standard deviations of differences of every fraction results; the listed Max of daily dose differences are the maximum of maximum differences of every fraction results. The listed Avg, Stdev, and Max normalized DDA differences are average, standard deviation, and maximum normalized DDA differences per fraction, respectively.

Significant differences were highlighted (grayed).

### 3.2 Dose differences of clinical constraints

Dosimetric constraint evaluation results are listed in Table 2. Constraint results from DDA based on two image reconstruction algorithms are compared with the constraint results of initial plans. Table 2 shows that DDA results could be very different from initial plans for PTV and sensitive structures including rectum, bladder, and penile bulb while results of both femur heads are similar. As highlighted in Table 2, D50% of rectum slightly exceeded the dose constraint of 40 Gy for patients PC09 and PC16. For all patients, differences of PTV Dmin due to CBCT reconstruction algorithms were −0.62 ± 4.60 Gy with a maximum difference of 8.31 Gy as highlighted in Table 2 and differences of CTV Dmin were −0.36 ± 0.84 Gy with a maximum difference of 1.87 Gy. Constraint differences for sensitive structures were 0.70 ± 1.49 Gy with a maximum of 5.68 Gy for penile bulb Dmean as highlighted for patient PC09.

**TABLE 2:** Dosimetric constraint results from initial Plan, DDA from FDK_CBCT, and DDA from iter_CBCT (iterative_CBCT) for 10 prostate patients

Patients			PC09			PC12			PC13			PC16			PC17			PC22			PC25			PC30			PC31		
Prescription			2GyX39 = 78 Gy			2GyX39 = 78 Gy			2 Gy X 39 = 78 Gy			1.8Gyx44= 79.2 Gy			1.8Gyx44= 79.2 Gy			1.8Gyx25 = 45 Gy			2GyX39 = 78 Gy			2GyX39 = 78 Gy			1.8Gyx44n= 79.2 Gy		
ROI	Specifi‐ cation	Goals	Plan	FDK_ CBCT	iter_ CBCT	Plan	FDK_ CBCT	iter_ CBCT	Plan	FDK_ CBCT	iter_ CBCT	Plan	FDK_ CBCT	iter_ CBCT	Plan	FDK_ CBCT	iter_ CBCT	Plan	FDK_ CBCT	iter_ CBCT	Plan	FDK_ CBCT	iter_ CBCT	Plan	FDK_ CBCT	iter_ CBCT	Plan	FDK_ CBCT	iter_ CBCT
PTV	D_mean_	>100% D_Rx_	79	79	79	81	81	80	80	78	78	81	80	80	81	78	78	45	44	44	79	79	79	79	79	79	81	81	81
PTV	D_min_	>95% D_Rx_	72	55	60	75	58	50	74	58	57	73	56	57	73	55	55	39	30	31	68	68	60	72	61	59	70	58	64
PTV	D_95%_	>95% D_Rx_	77	68	68	79	76	76	77	74	74	77	73	73	78	70	69	43	42	42	77	76	76	77	75	75	78	75	75
PTV	D_max_	<110% D_Rx_	82	80	80	83	83	83	83	81	81	86	84	84	83	82	82	46	46	46	82	81	81	82	80	80	85	84	84
CTV	D_min_	>95% D_Rx_	73	73	74	78	80	79	78	76	76	78	66	64	78	78	78	43	43	41	76	77	77	78	78	78	79	78	79
CTV	D_95%_	>95% D_Rx_	77	77	78	78	81	81	79	77	77	80	74	75	79	79	79	44	44	42	77	78	78	78	79	79	80	80	80
CTV	D_max_	<110% D_Rx_	81	80	80	82	83	83	82	80	80	85	83	83	83	81	81	46	45	46	81	81	81	80	80	80	84	84	84
Rectum	D_50%_	<40 Gy	37	40	41	32	33	32	34	36	36	40	42	42	35	37	37	25	25	25	27	31	30	37	36	36	38	40	40
Rectum	D_15%_	<75 Gy	68	61	62	64	66	64	55	55	56	73	70	69	60	53	53	37	37	37	55	58	58	69	67	68	71	70	70
Bladder	D_50%_	<50 Gy	11	12	16	27	39	39	27	29	30	45	43	46	24	28	32	25	26	26	33	32	33	47	50	50	46	48	48
Lt Femur Head	D_5%_	<50 Gy	38	36	35	36	37	37	25	25	25	NA	NA	NA	29	28	28	22	23	23	47	47	47	NA	NA	NA	45	46	46
Rt Femur Head	D_5%_	<50 Gy	30	31	30	29	30	30	26	26	26	NA	NA	NA	31	31	31	22	23	23	45	45	45	NA	NA	NA	43	44	44
Penile bulb	D_mean_	<50 Gy	49	42	48	42	40	41	10	9	9	51	48	51	22	20	24	NA	NA	NA	61	60	63	45	48	50	7	7	7

DDA were projected to full course based analyzed fractions of treatment results. Total prescription doses of full treatment courses are listed. Maximum and minimum doses are based on a small volume of 0.035 cc instead of a voxel. Dose unit is Gy.

Significant differences were highlighted (grayed).

### 3.3 DIR result differences due to CBCT reconstruction algorithms

**FIGURE 3:**
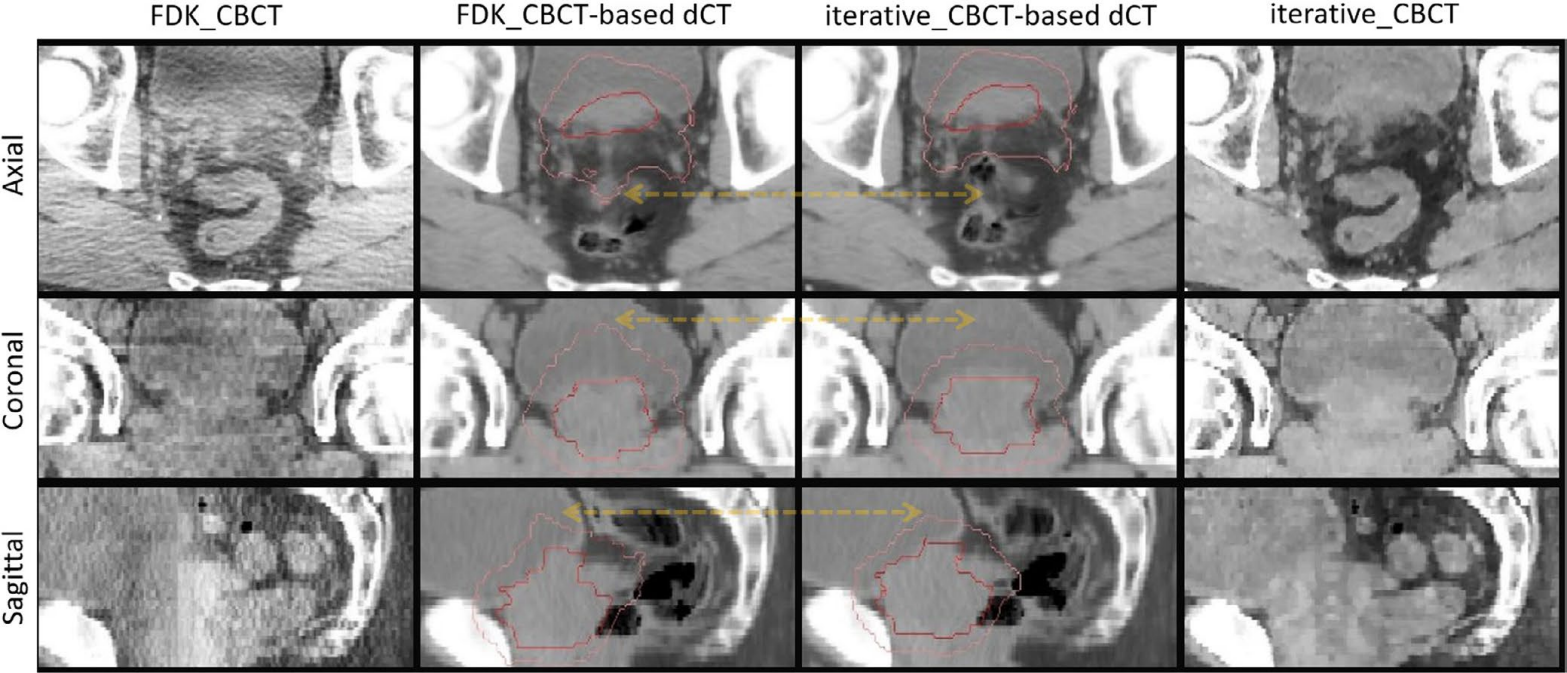
Axial (top row), coronal (middle row), and sagittal (lower row) views of FDK‐CBCT (left column) and iterative‐CBCT (right column) and associated deformed CT (dCT) registered from plan‐CT. Red arrows indicate image variations. HU# [‐300 300].

Figure 3 illustrates DIR differences between registration results using FDK_CBCT versus iterative_CBCT.

**FIGURE 4:**
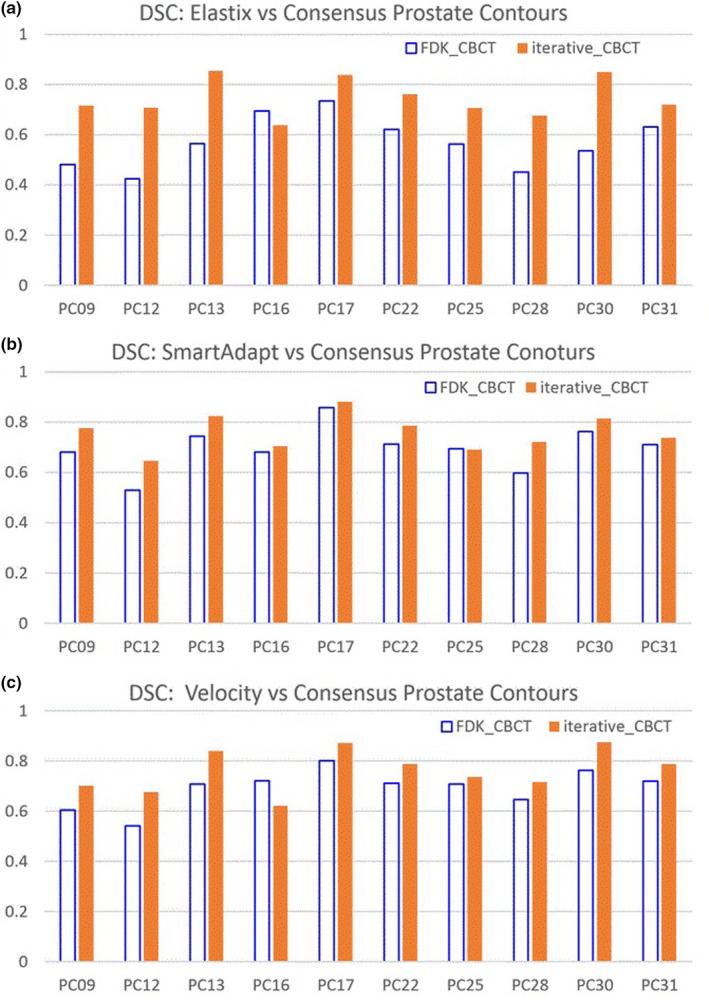
DSC between mapped and consensus prostate contours for three DIR algorithms: (a) Elastix; (b) SmartAdapt; and (c) Velocity.

Mapped contours from three DIR algorithms were compared against the consensus contours for each set of CBCT images. Figure 4 illustrates the DSC between mapped and consensus prostate (CTV) contours. The DSC increased from FDK_CBCT to iterative_CBCT by 0.18 ± 0.11, 0.06 ± 0.04, and 0.07 ± 0.07, for Elastix, SmartAdapt, and Velocity DIR algorithms respectively (*p* < 0.005). DSC improvement of all four volumes were listed for the three DIR algorithms in Table 3.

**TABLE 3:** DSC differences for three DIR algorithms

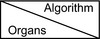	Elastix			SmartAdapt			Velocity		
	Average	Stdev	*p*‐value	Average	Stdev	*p*‐value	Average	Stdev	*p*‐value
Bladder	0.04	0.04	0.007	0.01	0.03	0.100	0.02	0.02	0.003
Prostate	0.18	0.11	0.0005	0.06	0.04	0.001	0.07	0.07	0.005
Rectum	0.06	0.04	0.001	0.01	0.04	0.245	0.02	0.03	0.020
SV	0.20	0.23	0.012	0.06	0.08	0.024	0.08	0.14	0.057

DSC difference = DSC(V_map_iterative_, V_cons_iterative_) ‐ DSC(V_map_FDK_, V_cons_FDK_). Average and Standard deviation (Stdev) of the DSC difference are listed. The statistical significance confidence level of DSC difference (*p*‐value) is listed.

**FIGURE 5:**
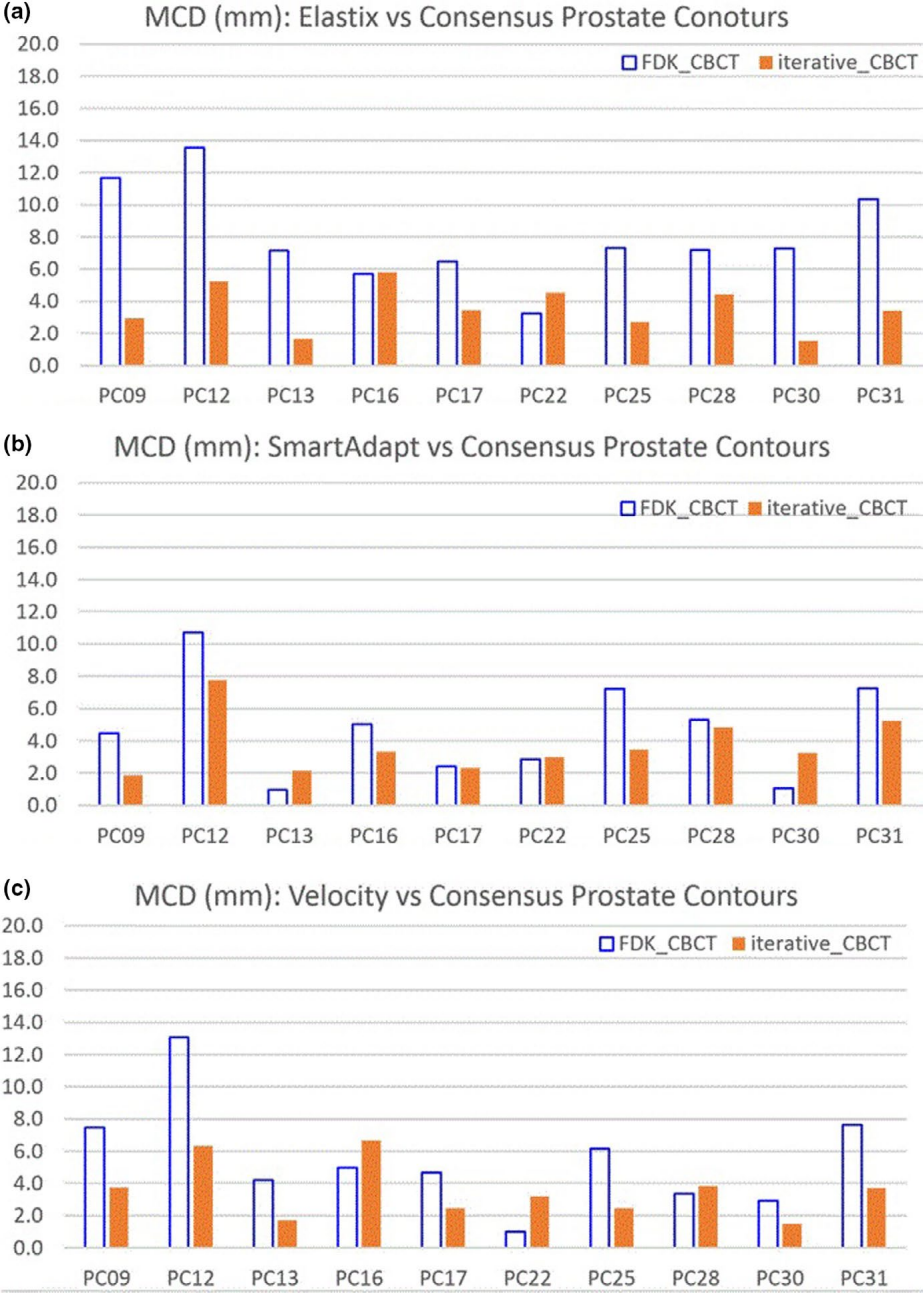
Mean Contour Distance (MCD) between mapped and consensus prostate contours for three DIR algorithms: (a) Elastix; (b) SmartAdapt; and (c) Velocity.

Figure 5 illustrates the MCD between mapped and consensus prostate contours. MCD difference results of all four contours were listed for three types of DIR algorithms in Table 4. Negative MCD differences indicate MCD reduction. The MCD of prostates was reduced from FDK_CBCT to iterative_CBCT by 4.4 ± 3.3 mm, 1.0 ± 1.9 mm, and 2.0 ± 2.8 mm, for Elastix, SmartAdapt, and Velocity DIR algorithms respectively.

**TABLE 4:** Mean Contour Distance (MCD) differences for organs for three DIR algorithms

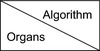	Elastix			SmartAdapt			Velocity		
	Average (mm)	Stdev (mm)	*p*‐value	Average (mm)	Stdev (mm)	*p*‐value	Average (mm)	Stdev (mm)	*p*‐value
Bladder	−0.1	2.9	0.474	−0.6	1.4	0.118	−0.3	1.5	0.280
Prostate	−4.4	3.3	0.001	−1.0	1.9	0.064	−2.0	2.8	0.025
Rectum	−1.8	2.7	0.034	−0.8	2.2	0.138	−1.0	1.2	0.014
SV	−3.6	8.4	0.103	−0.9	2.9	0.176	−2.7	3.2	0.014

MCD difference = MCD(V_map_iterative_, V_cons_iterative_) ‐ MCD(V_map_FDK_, V_cons_FDK_). Average and standard deviation (Stdev) of the MCD reduction were listed. The statistical significance confidence level of the MCD reduction (*p*‐value) is listed.

**FIGURE 6:**
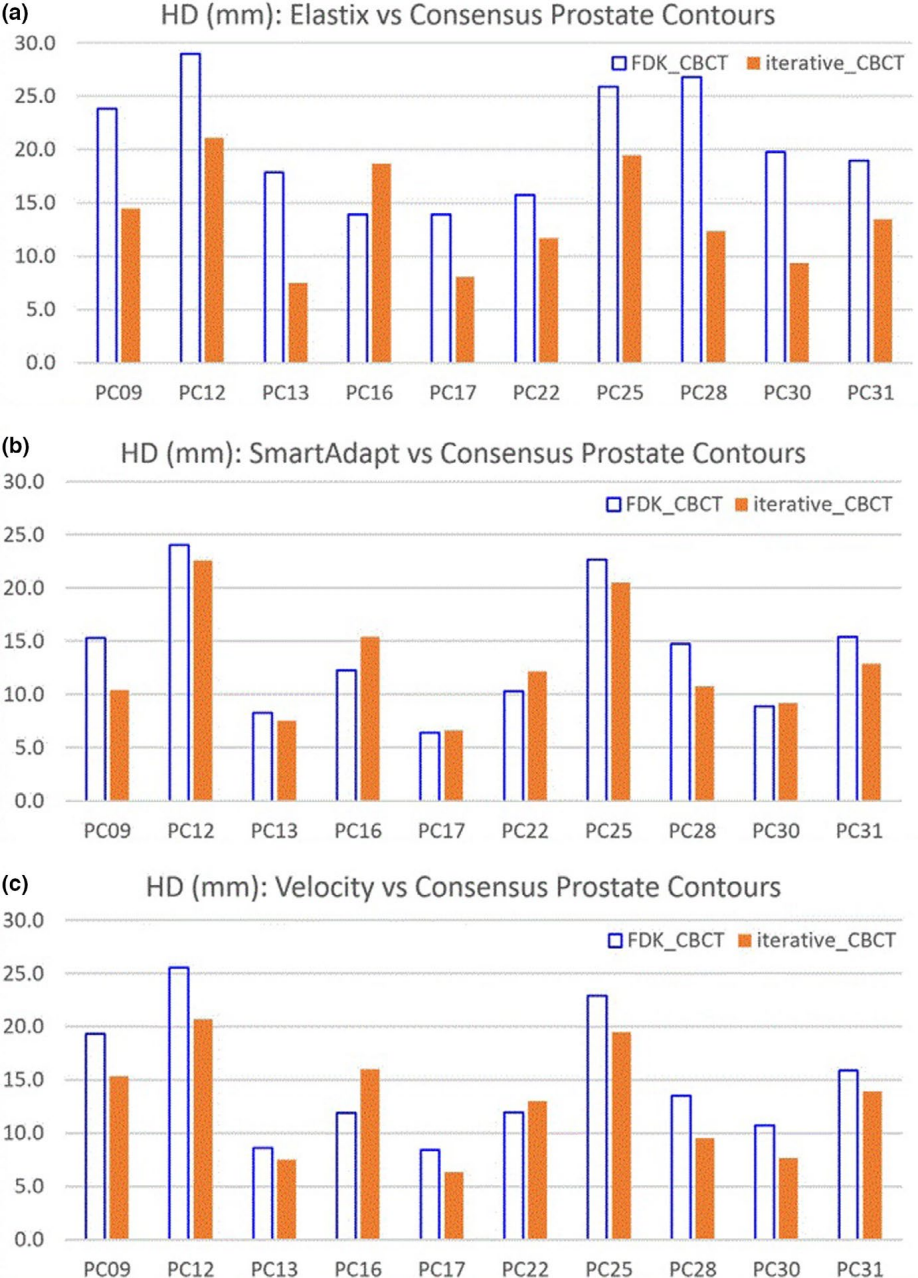
Hausdorff distance (HD) between mapped and consensus prostate contours for three DIRalgorithms: (a) Elastix; (b) SmartAdapt; and (c) Velocity.

Figure 6 illustrate the HD between mapped and consensus prostate contours. Results of HD difference between FDK_CBCT and iterative_CBCT were listed for three DIR algorithms in Table 4. Negative HD differences indicate HD reduction. The HD was reduced from FDK_CBCT to iterative_CBCT by 6.9 ± 5.1 mm, 1.0 ± 2.5 mm, and 1.9 ± 2.7 mm, for Elastix, SmartAdapt, and Velocity DIR algorithms respectively.

## 4 DISCUSSION

One big challenge of DDA study is that it lacks a ground truth. This study tried to compare dose differences due to CBCT image reconstructions at different processing stages: daily dose differences before dose mapping, daily dose differences after dose mapping, and normalized DDA differences. Both Table 1 and Figure 2 indicate that daily dose distributions are almost identical on dCT based on CBCT reconstructed by either algorithm. Two dCT sets have almost same bony structures (as shown in Figure 3) and external boundary. Their internal soft tissue differences are not significant enough to affect dose distributions of the same treatment plan delivery. However, after dose mapping from dCT to simCT, maximum daily dose differences increased from 0.22 ± 0.06 Gy to 1.33 ± 0.38 Gy for 323 fractions of treatments. Given that the daily prescribed dose is 1.80 Gy or 2.00 Gy, the dose mapping leads to significant dosimetric differences. As shown in Figure 2, large dose differences occur on the boundary of the target, where dose gradient is the highest. In these regions, minor image registration deviations may lead to significant dose differences. This is not occurred randomly since dose accumulation does not smear such differences completely. The maximum differences of normalized DDA are 0.42 ± 0.19 Gy, which are less than the maximum daily dose differences after dose mapping. However, maximum differences of normalized DDA are the average cumulative differences per fraction and random deviations are likely to be smeared over a course of treatment. The total DDA differences are differences of normalized DDA multiplied by number of fractions, ranging from 25 to 44. As a result, total maximum differences of DDA were 20.68 Gy and 16.13 Gy for patient PC09 and PC25 respectively. This means that total dose differences after dose mapping are significant. DDA presents actual total delivered dose and defines final clinical constraint results. Dosimetric constraint results confirmed the notable differences. As shown in Table 2, PTV could lose coverage over a treatment course as Dmin could be reduced from 75 Gy (patient PR12) to 58 Gy (based on FDK_CBCT) or 50 Gy (based on iterative_CBCT). It is understood that a small shape change of PTV may result in a cold spot near its surface and this might not be compensated by other fractions of treatment.^8^ The differences in PTV Dmin (up to 8 Gy due to CBCT reconstruction algorithm differences) are worthwhile to investigate the effects on deformable dose accumulation.

Because dose mapping uses deformable image registration results, the above analysis indicates that image registration results have been affected by different CBCT reconstruction algorithms. As shown in previous studies, patient image quality and soft‐tissue contrast are improved in the pelvis when using iterative CBCT.^17^ Image quality in the pelvis region surrounding the target, prostate, appears to be substantially better with the iterative CBCT reconstruction algorithm. Iterative_CBCT images have better low contrast detection in the pelvis than FDK_CBCT images. This will lead to more accurate and more reliable image registration for iterative_CBCT than FDK_CBCT images for prostate patients. At the same time, this region is also in the high dose gradient region, where registration results affect deformed doses to a much greater extent. This is likely why daily dose differences for prostate patients increased by five times after dose mapping and DDA dose differences were up to 31.2 Gy (for a course of 39 fractions for patient PC09). This also could explain why CTV dose coverage was affected much less than PTV dose coverage. As listed in Table 2, PTV Dmin differences were −0.62 ± 4.60 Gy for 10 patients, while CTV Dmin differences were −0.36 ± 0.84 Gy and CTV Dmax differences were 0.06 ± 0.17 Gy. Dose gradient is low around CTV and dose mapping differences will not affect CTV dose notably. At the same time, dose constraint results of both femoral heads were not affected by CBCT reconstruction algorithms since iterative_CBCT has minimal effects on bony structure results and femoral heads are normally well‐positioned for treatment.

This study was focused on clinical effects of image quality improvement due to iterative_CBCT reconstruction on deformable image registration instead of technical aspects of image registration. Contour mapping was compared with manual contours. One pair of CBCT images per prostate patient were manually contoured by three to five physicians for the purposes of generating a consensus contour, which was used as the ‘ground truth’ contour. DSC indicates the similarity between two volumes. DSC between prostate mapped contours and consensus contours increased for 9 of 10 patients, and for all three DIR algorithms increases on average by 0.10 ± 0.09 from FDK_CBCT results to iterative_CBCT results (*p* < 0.00001). MCD indicates the distance between centers of two volumes. Prostate contour MCD for three DIR algorithms was reduced by 2.5 ± 3.0 mm from FDK_CBCT results to iterative_CBCT results (*p* < 0.00005). This means that centers of mapped prostate contours moved closer to those of consensus prostate contours from FDK_CBCT results to iterative_CBCT results. HD indicates the extreme differences of contours. HD between mapped prostate contours and consensus prostate contours decreased by 3.3 ± 4.4 mm from FDK_CBCT results to iterative_CBCT results (*p* < 0.00015). All results show that mapped contours based on iterative_CBCT images were similar and closer to consensus contours than those based on FDK_CBCT images.

Three different deformable image registration methods were applied to eliminate their effects or sensitivities on image quality. Elastix and Velocity are B‐spline‐based while SmartAdapt is Demons‐based. Figures 4, 5, 6 and Tables 3, 4, 5 show that the three different DIR algorithms behave differently. Elastix DIR leads to larger differences due to CBCT reconstruction algorithms than either SmartAdapt or Velocity DIR regarding DSC, MCD, and HD in general. For the prostate contours, from FDK_CBCT to iterative_CBCT, improvement of DSC, MCD, and HD are twice or more are observed for Elastic DIR than either SmartAdapt or Velocity DIR. This indicates that DIR methods do have different sensitivities to image quality. All dosimetric studies in this report are based on Elastix DIR and this may also explain why significant dosimetric differences occur with Elastix DIR after dose mapping.

**TABLE 5:** Results Hausdorff distance (HD) difference for organs for three DIR algorithms

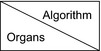	Elastix			SmartAdapt			Velocity		
	Average (mm)	Stdev (mm)	*p*‐value	Average (mm)	Stdev (mm)	*p*‐value	Average (mm)	Stdev (mm)	*p*‐value
Bladder	−2.7	3.3	0.014	−2.1	2.3	0.009	−1.0	2.1	0.088
Prostate	−6.9	5.1	0.001	−1.0	2.5	0.112	−1.9	2.7	0.025
Rectum	−4.4	6.6	0.033	−4.4	6.6	0.033	−2.9	5.2	0.054
SV	−8.7	9.2	0.007	−8.7	9.2	0.007	−4.0	6.0	0.032

HD difference = HD(V_map_iterative_, V_cons_iterative_) ‐ HD (V_map_FDK_, V_cons_FDK_). Average and Standard deviation of HD difference were listed. The statistical significance confidence level of HD difference (*p*‐value) is listed.

It should be noted that several factors may affect the evaluation of deformable image registration in addition to CBCT image quality differences due reconstruction algorithms. The structures mapped from contours originally based on fan‐beam CT, simCT, which has better image quality than either CBCT and this may lead to different contours on simCT and consensus contours on CBCT. More importantly, patient anatomies may be different every day due to bladder/colon/rectum filling status or response to radiation therapy. DIR is trying to match simCT to daily CBCT but the ground truth may be questionable. It may not be concluded that iterative_CBCT would lead to better DIR than FDK_CBCT for all structures since some structures changed after simCT was acquired and none of current DIR methods could completely match it. As displayed in Figure 3, there are air bubbles in colon during simCT acquisition but they disappeared in the day when CBCT was acquired. However, better soft tissue visibility leads to better DIR results or easier delineation for certain structures with less daily changes, like prostate. This is consistent with previous studies too.^17^ This is very valuable for dose accumulation of prostate cancer treatment.

## 5 CONCLUSIONS

Iterative_CBCT‐based image datasets lead to different mapped volumes of interest, deformed and cumulative doses than results based on conventional FDK_CBCT. This is particularly important for prostate cancer cases since better soft tissue visibility afforded with iterative_CBCT improves image registration accuracy at certain organs including prostate in the regions of high dose gradients. The improved image quality from iterative_CBCT is a promising approach to potentially yield more accurate DIR and adaptive accumulated dose distributions relative to FDK_CBCT.

## CONFLICT OF INTEREST

This work was supported in part by a grant from Varian Medical Systems, Palo Alto, CA.

## Data Availability

The data that support the findings of this study are available from the corresponding author upon reasonable request.
